# Effects of a strength physical exercise program in chronic lymphocytic leukemia patients on quality of life, mental health, and frailty: a randomized controlled trial study protocol

**DOI:** 10.3389/fspor.2025.1534861

**Published:** 2025-03-10

**Authors:** Juan Luis Sánchez-González, Eduardo José Fernández-Rodríguez, Roberto Méndez-Sánchez, Luis Polo-Ferrero, Ana Silvia Puente-González, Cristina de Ramón, Sara Marcos-Asensio, Patricia Blázquez-Benito, Almudena Navarro-Bailón, Fermín Sánchez-Guijo, Carlos Martín-Sánchez

**Affiliations:** ^1^ Department of Nursing and Physiotherapy, Universidad de Salamanca, Salamanca, Spain; ^2^ Institute of Biomedical Research of Salamanca (IBSAL), Salamanca, Spain; ^3^ Hematology Department, Hospital Universitario de Salamanca, Salamanca, Spain; ^4^ Faculty of Medicine, University of Salamanca, Salamanca, Spain; ^5^ Biomedical Research Networking Center for Cancer (CIBERONC), ISCIII, Salamanca, Spain

**Keywords:** exercise, chronic lymphocytic leukemia, cancer, physiotherapy, protocol

## Abstract

**Background:**

Chronic lymphocytic leukemia (CLL) is a type of cancer that affects the blood and bone marrow, and it is the most prevalent form of leukemia in adults. Many patients experience symptoms that can significantly impact their quality of life, particularly in terms of physical ability, emotional health, and fatigue. Therapeutic exercise has shown to be an effective intervention for alleviating both physical and psychological symptoms in these patients. Specifically, strength training may help address some common treatment side effects.

**Objective:**

This study aims to evaluate the effects of a therapeutic exercise program, focused on frailty in patients with CLL, along with secondary objectives including impacts on functional capacity, quality of life, psychological status, sleep quality, body composition, anthropometric variables, lipid profile and on proteins related to the immune system and inflammation.

**Methods:**

An open label, randomized controlled trial will be carried out with 36 participants, divided into an intervention group (supervised resistance training twice a week and home exercises) and a control group (home exercise only). The primary outcome measure is fraility, assessed using Short Physical Performance Battery (SPPB). Secondary outcomes include assessments using HADS, FACT-F, EORTC QLQ-C30, EORT QLQ-CLL17.

**Results and conclusions:**

This study will explore how physical exercise can improve quality of life and various health metrics in patients with CLL. By creating customized exercise protocols, the research seeks to boost patient well-being, improve treatment outcomes, and lessen debilitating side effects, ultimately promoting the integration of physical activity into routine care.

**Clinical Trial Registration:**

ClinicalTrials.gov, identifier (NCT06654206).

## 1 Introduction

Chronic lymphocytic leukemia (CLL) is a malignant hematological disease that mainly affects older people, with a mean age at diagnosis of about 70 years, and is the most common form of leukemia in adults (1, 2). It has an incidence of 4–5 cases per 100,000 people in Western countries, accounting for nearly 30% of all adult leukemias (3). This condition is characterized by an accumulation of mature B lymphocytes in the blood, bone marrow, and lymph nodes. Its progression is generally slow, though the clinical development can vary: some patients remain stable for years without needing treatment, while others require early medical intervention (4).

As CLL progresses, many patients experience symptoms that affect their quality of life, especially regarding functional capacity and emotional well-being. Fatigue is one of these most characteristic symptoms (5), and directly correlates with muscle weakness, lack of energy, and a consequent loss of physical functionality, leading to limitations in daily activities, reduced mobility, and, ultimately, a loss of autonomy and social interaction (6). Additionally, side effects from CLL treatments, such as BTK inhibitors, BCL2 inhibitors and monoclonal antibodies can exacerbate this functional decline, creating a cycle of physical and emotional limitations that severely impact quality of life.

The frailty phenotype involving five domains [unintentional weight loss (10 lbs in past year), self-reported exhaustion, weakness (grip strength), slow walking speed, and low physical activity], and characterizes physical frailty as a subset of frailty (7). In turn, sarcopenia was redefined in 2019 by EWGSOP2 (8) and is now considered a syndrome characterized by progressive and generalized decline in muscle mass, strength and function, associated with age and leading to adverse events. Considering this, physical frailty and sarcopenia are strongly related and sarcopenia has been described as the biological substrate of physical frailty (9).

The emotional aspect also plays a crucial role in CLL patients. Literature suggests that the diagnosis and course of this chronic disease generate high levels of stress, anxiety, and depression—emotions that affect perceived control over the disease and the adaptation to the diagnosis (10). This emotional burden not only worsens other physical symptoms but can increase fatigue and reduce motivation to participate in physical and social activities.

In light of these clinical challenges, therapeutic exercise has proven to be a promising intervention for improving both physical and psychological symptoms in these patients (11–15). Specifically, strength training could help manage some of the common adverse effects of CLL. Strengthening muscles and increasing muscle mass helps counteract weakness and improve functional capacity, promoting independence in daily activities and reducing feelings of fatigue (16). Additionally, strength training could improve bone health, joint stability, and balance, which are often compromised in CLL patients, increasing the risk of falls and fractures (17).

Furthermore, recent pilot studies have shown that different types of exercise can be beneficial for patients with chronic lymphocytic leukaemia. Moderate-intensity aerobic activity can improve cardiorespiratory fitness, reduce chronic fatigue and strengthen the immune system (18) and high-intensity interval training (HIIT) with strength training had strong effects on muscle strength and immune function in previously untreated older adults with CLL (19), all of which are crucial for patients with haematological diseases such as CLL. In this regard, a training intensity of 70% of the 1-RM with 12 repetitions has been selected, as previous studies have indicated that lower intensities may not provide a sufficient stimulus to counteract sarcopenia and the functional decline associated with the disease and its treatments (20). Therefore, this approach has been considered optimal for maximizing benefits without compromising patient safety.

It is also hypothesized that strength training could be particularly beneficial given the clinical characteristics of the disease. CLL is characterized by immune dysfunction and a high risk of sarcopenia and bone density loss, which can significantly reduce patients’ physical functionality. Muscle strengthening through resistance training could mitigate these effects, contributing to improved muscle and bone health. Furthermore, strength training could reduce the risk of falls and fractures, which are common issues in this population.

On the other hand, physical activity appears to have positive emotional effects, including reductions in anxiety and depression, which promotes better sleep (21). Beyond its physiological benefits, regular exercise contributes to improved self-esteem and self-perception, offering patients a sense of control over their health. This is especially relevant in chronic diseases like CLL, where patients often feel a loss of control (22). Thus, exercise could not only improve quality of life but also provide a tool for facing emotional challenges related to disease, particularly relevant consideration in a long-term illness.

Despite this evidence, there is still limited research on the benefits of strength training in cancer patients, and specific studies on the effects of strength training programs in CLL are particularly lacking. Most studies have focused on more prevalent cancers, such as breast or prostate cancer, leaving a gap in knowledge about the impact of exercise on hematological diseases like CLL. Specifically, studies exploring how strength training influences specific aspects of health in these patients are needed.

In this context, our study aims to contribute to the existing knowledge by evaluating the effects of a therapeutic exercise program focused on strength training on general functional capacity and quality of life in CLL patients. That is why we propose a study protocol that addresses all these clinical challenges in this type of patients, aiming to help establish evidence-based clinical intervention protocols in the future.

As the primary objective, we aim to evaluate the effects of a supervised therapeutic exercise program, focused on strength training, on frailty in patients undergoing treatment for CLL. And as secondary objectives, the following:

• To characterize and describe the functional and psychological status of participants treated for CLL.
• To assess the impact of a therapeutic strength-based exercise program on quality of life in participants under treatment for CLL.
• To assess the impact of a therapeutic strength-based exercise program on sleep quality in participants under treatment for CLL.
• To evaluate the impact of a therapeutic strength-based exercise program on specific aspects of functional physical capacity in participants treated for CLL, including fatigue, upper and lower limb strength and walking speed.
• To assess the impact of a therapeutic strength-based exercise program on psychological well-being in participants with treatment for CLL, with a focus on anxiety and depression.
• To evaluate the impact of a therapeutic strength-based exercise program on body composition and other anthropometric variables in participants under treatment for CLL.
• To assess the impact of a therapeutic strength-based exercise program on lipid profile and on proteins related to the immune system and inflammation.

## 2 Methods and analysis

### 2.1 Study design and setting

An open-label, randomized controlled trial will be carried out at the University of Salamanca's Faculty of Nursing and Physiotherapy (Spain), adhering to the Consolidated Standards of Reporting Trials (CONSORT) guidelines (23). The current treatment protocol is detailed based on SPIRIT recommendations (24). A detailed overview of participant enrolment, interventions, and assessments is provided in the Supplementary Material (Supplementary Table S1). The Research Ethics Committee for Medicinal Products of the IBSAL-Hospital Universitario de Salamanca has approved the trial protocol (record number2024/11), and the study will be conducted in compliance with the Declaration of Helsinki. This clinical trial has been registered on ClinicalTrials.gov (NCT06654206).

### 2.2 Participants and eligibility criteria

Participants diagnosed with Chronic Lymphocytic Leukemia (CLL) undergoing active treatment will be recruited from the Hematology Department of the Hospital Universitario de Salamanca.

The inclusion criteria are as follows:

• Participants diagnosed with CLL or Small Lymphocytic Lymphoma (SLL) undergoing active treatment.
• Participants who have not engaged in regular physical activity in the past 8 weeks.
• Performance Status (ECOG) 0–1.
• Signed informed consent.

The exclusion criteria are:

• The presence of any medical contraindications to physical exercise, such as severe musculoskeletal disorders, serious cardiovascular diseases, bone metastases, or other conditions as determined by a healthcare professional.
• Participants unable to complete the initial evaluation tests or who have difficulty performing basic exercises.
• Other circumstances, as determined by the researchers, that may interfere with the study's objectives or development.

Participants will be discontinued if they meet any of the following:

• Negative developments or progression of the tumor process.
• Severe adverse events related to exercise.
• Voluntary withdrawal from the intervention.
• New medical contraindications that prevent safe participation.
• Non-adherence to the intervention protocol that compromises study integrity.

These criteria ensure participant safety and adherence to ethical research guidelines.

### 2.3 Intervention

The exercise intervention program has been tailored for participants diagnosed with CLL undergoing active treatment. To ensure appropriate methodological quality and replicability of the exercise program, the Consensus on Exercise Reporting Template (CERT) (25) guidelines will be followed for a detailed description of the intervention (Supplementary Table S2). It primarily emphasizes resistance training, while also incorporating daily aerobic activities. This intervention will span a duration of 8 weeks. The study sample will be randomly split into two groups: the intervention group and the control group, as described below.

#### 2.3.1 Intervention group

The intervention group will engage in a supervised resistance training program twice a week, along with a home-based physical activity promotion program to be completed three times a week. All supervised training sessions will be led by a physiotherapist at a healthcare facility.

##### 2.3.1.1 Supervised training sessions

There will be two weekly sessions, each one lasting 50 min. Each session will have three distinct parts:

- Warm-up: Begin with 10 min of full-body strength and aerobic exercises focusing on the muscle groups that will be targeted in the upcoming session. Exercise intensity will be monitored using the 0–10 Rating of Perceived Exertion (RPE) scale, following the recommendations of the American College of Sports Medicine (26), participants will be required to maintain a perceived effort of at least 6 on the RPE scale.
- Resistance Training: Participants will perform six resistance exercises targeting the major muscle groups over a 30-minute period. The initial load will be set at 70% of the estimated one-repetition maximum (1-RM). When a participant successfully completes three sets of 12 repetitions at this weight for two consecutive sessions, the load will be increased by 10%. The exercises will follow a circuit format, with 30 s of rest between exercises and 90 s of rest between sets. Exercise intensity will be monitored using the 0–10 RPE scale, following the recommendations of the American College of Sports Medicine. To ensure an adequate training stimulus, participants will be required to maintain a perceived effort of at least 7 on the RPE scale. This approach allows for a precise regulation of exercise intensity, considering individual variations in training response and ensuring sufficient overload for adaptation. For exercises that include jumps, such as Sit-to-Stand Jumps, Single-Leg Box Step-Ups, and Jump Squats, only body weight will be used initially. These exercises will not progress based on 1RM but will be adjusted following RPE, ensuring that participants maintain an appropriate intensity while minimizing the risk of injury
- Cooldown/Mobility: Finish with 10 min of a combination of deep breathing exercises and global mobility work to promote recovery.

The following describes the four training sessions, which will be repeated in order and cyclically until the 8 weeks of training are completed.

###### 2.3.1.1.1 Session 1

1. Warm-up (10 min)
  • 2 min: Walk in circles taking advantage of the width of the room incorporating mobility exercises.
    ○ 20 s: Just walk fast.
    ○ 20 s: Walk with a long stride.
    ○ 20 s: Shoulder circles backward.
    ○ 20 s: Alternating knee lifts to chest.
    ○ 20 s: Walk on tiptoes.
    ○ 20 s: Lateral movement with jogging.
  • 2 min: Single-leg balance with slight knee and hip flexion, 1 min on each leg.
  • 1 min: Wall/floor push-ups.
  • 1 min: Wall sit.
  • 2 min: Skipping.
  • 2 min: Partner ball throws or wall throws.
2. Resistance Training
  • Bodyweight squat.
  • Romanian deadlift.
  • Abdominal plank (3 set of 30 s).
  • Bench press with barbell.
  • Bicep curls dumbbells + shoulder flexion.
  • Dumbbell rows.

*There will be 6 stations, and participants will rotate from one to the next until completing all 6 exercises. They will do 3 sets of 12 repetitions at 70% of 1RM. For unilateral exercises, 3 sets of 12 repetitions will be performed on each side. Rest 30 s between stations and at the end of each complete set, rest for 90 s.

3. Cool-down
  • 3 min: Slow-paced walking with diaphragmatic breathing.
  • 1 min: Quadriceps stretch.
  • 1 min: Hamstring stretch.
  • 1 min: Calf stretch.
  • 1 min: Bicep stretch.
  • 1 min: Shoulder stretch.
  • 2 min: Seated cervical mobility exercises.

###### 2.3.1.1.2 Session 2

1. Warm-up (10 min)
  • 2 min: Walk in circles taking advantage of the room's width incorporating mobility exercises.
    ○ 20 s: Just walk.
    ○ 20 s: Shoulder abduction/adduction in transverse plane.
    ○ 20 s: Alternating shoulder flexion/extension.
    ○ 20 s: Leg lifts with straight knees alternately.
    ○ 20 s: Walk on tiptoes.
    ○ 20 s: Jumping in place.
  • 2 min: Ankle proprioception on single-leg support, 1 min on each leg.
  • 1 min: Knee lift with contralateral arm raise.
  • 1 min: Bodyweight squats/sit and stand from a chair.
  • 2 min: Step-up with contralateral knee raise.
  • 2 min: Boxing (punching forward with alternate arms at a slow pace).
2. Resistance Training
  • Forward lunge with sandbag/dumbbell/kettlebell.
  • Hip thrust with barbell/kettlebell.
  • Pallof press with resistance band.
  • Dumbbell bench press.
  • Dumbbell shoulder press.
  • Sit-to-stand jumps.

*There will be 6 stations, and participants will rotate from one to the next until completing all 6 exercises. They will do 3 sets of 12 repetitions at 70% of 1RM. Rest 30 s between stations and at the end of each complete set, rest for 90 s.

3. Cool-down
  • 3 min: Slow-paced walking with diaphragmatic breathing.
  • 1 min: Quadriceps stretch.
  • 1 min: Glute stretch.
  • 1 min: Adductor stretch.
  • 1 min: Hamstring stretch.
  • 1 min: Shoulder stretch.
  • 2 min: Seated cervical mobility exercises.

###### 2.3.1.1.3 Session 3

1. Warm-up (10 min)
  • 2 min: Walk in circles taking advantage of the room's width incorporating mobility exercises.
    ○ 20 s: Just walk.
    ○ 20 s: Elbow flexion/extension.
    ○ 20 s: Shoulder abduction/adduction.
    ○ 20 s: Hip flexion + abduction.
    ○ 20 s: Walking lunges.
    ○ 20 s: Side steps.
  • 2 min: Lateral step-ups, 1 min on each leg.
  • 1 min: Close-grip push-ups on wall/floor.
  • 1 min: Bodyweight alternating lunges in place.
  • 2 min: Jumping jacks.
  • 2 min: Bodyweight glute bridges.
2. Resistance Training
  • Wide stance squat.
  • Deadlift.
  • Barbell rows.
  • Barbell bicep curls.
  • Dumbbell lateral shoulder raises.
  • Single-leg box step-ups.

*There will be 6 stations, and participants will rotate from one to the next until completing all 6 exercises. They will do 3 sets of 12 repetitions at 70% of 1RM. Rest 30 s between stations and at the end of each complete set, rest for 90 s.

3. Cool-down
  • 3 min: Slow-paced walking with diaphragmatic breathing.
  • 1 min: Quadriceps stretch.
  • 1 min: Glute stretch.
  • 1 min: Hamstring stretch.
  • 1 min: Bicep stretch.
  • 1 min: Shoulder stretch.
  • 2 min: Seated cervical mobility exercises.

###### 2.3.1.1.4 Session 4

1. Warm-up (10 min)
  • 2 min: Walk in circles taking advantage of the room's width incorporating mobility exercises.
    ○ 20 s: Just walk.
    ○ 20 s: Squat and stand up as you walk.
    ○ 20 s: Trunk rotations with shoulder push.
    ○ 20 s: Shoulder raises.
    ○ 20 s: Side lunges.
    ○ 20 s: Walk backward.
  • 2 min: Sit and stand from a chair.
  • 1 min: Theraband elbow flexion.
  • 1 min: Calf raises and holds statically.
  • 2 min: Jumping in place.
  • 2 min: Shoulder circumduction with light weight.
2. Resistance Training
  • Lateral lunge with kettlebell.
  • Deadlift.
  • Incline bench chest fly with dumbbells.
  • Supine triceps press.
  • Front shoulder raises with plate.
  • Jump squats.

*There will be 6 stations, and participants will rotate from one to the next until completing all 6 exercises. They will do 3 sets of 12 repetitions at 70% of 1RM. Rest 30 s between stations and at the end of each complete set, rest for 90 s.

3. Cool-down
  • 3 min: Slow-paced walking with diaphragmatic breathing.
  • 1 min: Quadriceps stretch.
  • 1 min: Glute stretch.
  • 1 min: Triceps stretch.
  • 1 min: Chest stretch.
  • 1 min: Shoulder stretch.
  • 2 min: Seated cervical mobility exercises.

##### 2.3.1.2 Physical activity promotion

Participants will complete three weekly home training sessions that will not coincide with supervised training days. Each session will last 20 min, they will complete two sets of five exercises, with one minute of work and one minute of rest.
- Day 1
  1. Sitting and standing up from a chair.
  2. Lateral lunge.
  3. Supine bicycle.
  4. Elbow flexion with weights.
  5. Lateral shoulder raises.
- Day 2
  1. Lunge.
  2. Quadriceps wall isometric.
  3. Glute bridge.
  4. Frontal shoulder raises.
  5. Wall push-ups.
- Day 3
  1. Walking on tiptoes.
  2. 2. Squats.
  3. Knee-to-chest raises.
  4. Abdominal crunch.
  5. Shoulder press with weights.

Additionally, all participants should walk for 60 min each day of the week.

#### 2.3.2 Control group

The control group will only perform the physical activity promotion program 5 days/week. This will be combined with walking for 60 min every day of the week.

### 2.4 Outcomes

During the initial assessment, all variables, including sociodemographic factors, will be measured. After the intervention period, all outcome variables will then be evaluated again.

Personal and sociodemographic variables are as follows:

• Age (years)
• Gender (male/female)
• Weight (kilograms), height (meters) and body mass index (kg/m^2^)
• Time since diagnosis (years and months)
• Diagnosis (CLL or small lymphocytic lymphoma)
• IGHV mutational status (mutated/unmutated)
• Number of prior lines of treatment
• Current treatment and dose
• Time under current treatment (months)
• Chromosome 17p deletion and *TP53* mutation status prior to current treatment (present/absent)
• Current treatment under clinical trial (yes/no)

#### 2.4.1 Primary outcomes

The primary outcome measure will be frailty: Frailty will be assessed using the Short Physical Performance Battery (SPPB) scale (27), which includes three components: a balance test, gait speed, and a chair stand test. The scale has a maximum score of 12 points, with higher scores reflecting better physical function.

#### 2.4.2 Secondary outcomes

The secondary outcomes are as follows:

• Quality of life: Quality of life will be evaluated using the European Organization for Research and Treatment of Cancer Quality of Life Questionnaire (EORTC QLQ). The EORTC QLQ-C30 is a widely used tool designed to assess quality of life in patients with cancer (28). Comprising 30 items, this questionnaire divides 24 items across five functional domains: physical, role, emotional, cognitive, and social. Additionally, it includes three symptom-related scales—fatigue, pain, and nausea/vomiting—and a global health status scale. Six further items evaluate other symptoms, including dyspnea, appetite loss, insomnia, constipation, and diarrhea, as well as financial burden. Patients respond to items using a four-point scale (“not at all”, “a little”, “quite a bit”, “very much”), except for the overall health and quality-of-life scales, which use a seven-point format. On the functioning and overall health and quality-of-life scales, higher scores reflect better health status. In contrast, higher scores on the symptom scales indicate a greater level of symptom burden. In addition, a specific questionnaire was used to measure quality of life in patients with CLL, the EORTC QLQ-CLL17 questionnaire. The EORTC QLQ-CLL17 includes 17 items, which are organized into three multi-item subscales: symptom burden from disease and/or treatment (six items), physical condition and fatigue (four items), and health-related worries or fears about functioning (seven items, with two being conditional). The conditional items pertain to work or education and are only rated by patients if relevant to their situation. Each item is rated on a four-point scale (“not at all”, “a little”, “quite a bit”, and “very much”), reflecting experiences over the past week (29). The scoring method for the QLQ-CLL17 follows that of the EORTC QLQ-C30, with items in each subscale summed and converted to a standardized scale from 0 to 100. Higher scores across all scales and individual items indicate greater levels of symptoms or issues.
• Anxiety and depression: Anxiety and depression will be evaluated using the Hospital Anxiety and Depression Scale HADS, a well-established and validated questionnaire (30) for various populations. HADS includes a total of 14 items, with seven questions dedicated to assessing anxiety (subscale A) and the other seven focused on depression (subscale D), each rated on a scale of 0–3. This structure provides separate scores for anxiety and depression.
• Fatigue: Fatigue will be evaluated using the 13-item Functional Assessment of Cancer Therapy-Fatigue (FACT-F) subscale (31), a self-reported tool designed to measure the severity of fatigue in cancer patients (32, 33). This widely used scale for assessing cancer-related fatigue combines 27 items from the FACT-General section with 13 additional items specific to fatigue, resulting in a score range from 0 to 160. Higher scores on the FACT-F indicate less fatigue.
• Sleep quality: Patient sleep quality will be assessed using the Athens Insomnia Scale (AIS), a self-administered tool consisting of eight items developed by Soldatos (34) and validated for use in the Spanish population (35). The first five items assess “sleep disturbances”—specifically, sleep onset, nocturnal awakenings, early morning awakening, total sleep duration, and overall sleep quality—corresponding to criterion “A” for insomnia diagnosis under the ICD-10 classification of mental and behavioral disorders. The last three items evaluate “daytime consequences”, such as daytime well-being, physical and mental functioning, and daytime sleepiness, aligned with criterion “C” of the ICD-10. Each item is rated on a Likert scale from 0 (“no problem”) to 3 (“significant problem”), yielding a total score range of 0–24. Higher scores reflect more severe impairment, with a score of ≥6 indicating the presence of insomnia (36).
• Physical activity: The overall physical activity will be measured with the Spanish version of the International Physical Activity Questionnaire (IPAQ) (37). The IPAQ includes seven questions designed to assess the duration and frequency of physical activities at different intensity levels: Light activity (<600 MET minutes/week), such as walking at home, at work, or leisure walking; Moderate activity (600–3,000 MET minutes/week), which includes activities like cycling, playing tennis, or carrying light loads; Vigorous activity (>3,000 MET minutes/week), involving high-intensity exercises such as aerobic workouts, digging, or heavy lifting. Additionally, the IPAQ evaluates inactivity over the past week, providing a comprehensive view of the participant's physical activity levels. The total PA score is the sum of vigorous, moderate, and light PA in MET minutes/week (38).
• Body composition: The collection of various variables related to the body composition of the participants will be carried out. Height will be measured using a measuring rod. The other variables will be obtained using the electrical bioimpedance technique, using the TANITA BC-418® device. Variables to be collected include total body weight and appendicular muscle mass (ASM), which is defined as the sum of the muscle mass of each limb. To calculate the appendicular muscle mass index (ASMI), the height-adjusted appendicular muscle mass formula (AMM/height^2^) will be applied (8). In addition, parameters such as visceral fat, body fat percentage (BF%) and body mass index (BMI) will be assessed. To ensure the accuracy of the data, all participants will be weighed with the minimum amount of clothing and barefoot, which minimizes variations in the measurements and ensures that the results are representative of their actual body composition (39).
• Strength: The strength of both the lower and upper limbs will be assessed. The Jamar® dynamometer (J00105 Lafayette Instrument Company, USA), a hydraulic dynamometer considered the gold standard in the evaluation of grip strength, will be used (40). Both hands will be measured. The measurement will be performed following the Southampton Protocol (41). The patient will be seated in a chair with a backrest and the test will be explained to them. Three consecutive measurements will be taken on the same hand with a duration of 5 s per contraction and with an interval of 10 s between measurements. Encouraging commands will be given during the test to squeeze harder. The patient shall be seated in a chair with backrest, shoulder adducted and neutrally rotated, elbow flexed at 90°, forearm in neutral position and wrist in slight extension (0°–30°). The second Jamar® position shall be selected for all participants, with the exception of small hands which shall be placed in the first position and reported. The Jamar® measurement shall always be performed by the same evaluator. The mean/maximum value of each hand will be selected for analysis. The assessment of lower extremity strength will be conducted through the Five-Repetition Sit-to-Stand Test (5STS), a functional test that quantifies the capacity to rise and sit from a chair without the use of the arms, thereby reflecting lower extremity strength. An armless folding chair with a seat height of 17 in (43.2 cm) will be utilized. The time taken to complete the five repetitions will be recorded from the moment the subject rises from the chair to the moment they return to the sitting position (42).
• Physical performance: In addition to the SPPB, walking speed is assessed as a measure of physical performance associated with frailty using the four-meter walk test (4MWT). The patient starts walking one meter before the start line, where the stopwatch is activated, and is instructed to maintain his or her usual speed until four meters past the finish line. The test is performed three times, and the best value is recorded (43).

### 2.5 Sample size

The sample size was determined based on functional capacity, assessed by the Short Physical Performance Battery (SPPB) with scores ranging from 0 to 12 points (27). According to previous research in subjects with similar characteristics, 1.34 points has been established as a substantial minimum demonstrable change, in order to avoid detection of small and irrelevant changes (44). Furthermore, in studies with interventions shorter than 12 weeks, with similar participants, the standard deviation of the SPPB has been estimated to range between 1 and 1.5 points (45, 46).

Considering these data, the sample size calculation to detect a difference equal to or greater than 1.34 points on the SPPB, with a standard deviation of 1.3 points, accepting an alpha risk of 0.05 and with a power of 80%, requires 18 participants in each group, also assuming a loss rate of 15%. This calculation was performed with GRANMO software version 7.12.

### 2.6 Allocation and randomization

The randomization process for this clinical trial will be conducted using Microsoft Excel 2020, which generated a list of random numbers for participant assignment. Each participant will be allocated a unique number from this list: participants with odd numbers were assigned to the experimental group, and those with even numbers to the control group. This method ensures a randomized and balanced distribution between groups, reducing potential bias and enhancing the validity of the trial results. This process will be carried out by a member of staff from the Haematology Department of the Hospital Universitario de Salamanca who is not involved in the research.

### 2.7 Blinding

Given the study design, blinding of participants and evaluators to the intervention will not be feasible. However, to maintain objectivity, statistical analysis will be performed by an independent statistician who is unaware of the participants' group assignments.

### 2.8 Statistical methods

For the descriptive analysis, data normality will be assessed using the Kolmogorov–Smirnov and Shapiro–Wilk tests (applicable for samples where *n* < 30). Variables following a normal distribution will be characterized by mean, standard deviation, and range, while non-normally distributed variables will be represented by median and interquartile range. Qualitative variables will be described through frequencies and percentages.

In terms of quantitative analysis, Pearson's correlation coefficient will be used to confirm the validity of the assessment procedures chosen for this study, and Cronbach's alpha will verify their reliability. For comparisons between two means, we will employ Student's *t*-test (a parametric test for independent samples), the Mann–Whitney *U*-test (a non-parametric test for two independent samples), and Wilcoxon's test (a non-parametric test for paired data). For comparisons across three or more means, an ANOVA will be used for independent groups (parametric with Snedecor's *F*-test and non-parametric with the Kruskal–Wallis *H*-test). For repeated measures, Snedecor's *F*-test will be applied to parametric data, while Friedman's test will be used for non-parametric data. Correlations will be analyzed using Pearson's correlation for normally distributed data and Spearman's correlation for non-normally distributed data.

To identify variables associated with outcomes of interest, a multivariate logistic regression analysis will be conducted. This model will include variables that are either significant in the bivariate analysis or pertinent to the study. For qualitative or categorical variables, contingency tables and the Chi-square test will be used for comparisons involving two independent samples.

*p*-value of less than 0.05 will be considered statistically significant, indicating a confidence interval of 95%. Analyses will be performed using IBM SPSS Statistics, version 28.0.1.

## 3 Discussion

### 3.1 Potential impact and significance of the study

The implementation of a strength-training exercise program for patients with CLL has significant practical and clinical implications that can transform the care and management of this disease. First and foremost, muscle strength and endurance play a crucial role in reducing frailty in patients with chronic lymphocytic leukemia (CLL), who, with a median diagnosis age of 71, are at high risk of developing this condition. Frailty is a clinical state that increases vulnerability to adverse events and can hinder recovery and adherence to medical treatments. Through a structured strength-training program, patients can improve their daily functionality, promoting independence and reducing the risk of falls and injuries. This not only enhances their physical well-being but can also lessen the burden on the healthcare system by reducing hospitalizations and related complications.

Additionally, implementing a strength-training exercise program for patients with CLL has significant clinical and practical implications, particularly in improving their quality of life. Regular physical activity can help mitigate treatment side effects such as fatigue and muscle weakness and is associated with greater emotional well-being and overall life satisfaction. By integrating strength training into CLL management, patients are provided with effective tools to cope with the physical and emotional challenges of the disease, contributing to a more comprehensive and effective approach to treatment.

The implications of exercise also extend to mental health. Research has shown that CLL patients may experience high levels **negative** of anxiety and depression, conditions that negatively affect their quality of life. A strength-training program can act as an effective mood regulator, helping to alleviate symptoms of anxiety and depression. By improving patients' self-efficacy and self-esteem through physical strengthening, mental and emotional health can be positively impacted. Furthermore, exercise has shown positive effects on sleep quality, a critical aspect that is often compromised in oncology patients. Improved sleep not only enhances physical health but also supports emotional well-being, creating a positive cycle of health.

In conclusion, this physical exercise program could provide substantial benefits for patients with CLL who are receiving pharmacologic therapy. It may enhance their quality of life, physical abilities, and potentially their clinical outcomes, while also fostering a positive mindset and encouraging healthy lifestyle habits. By comparing the anticipated results of this study with existing research, it is clear that this work will make a meaningful contribution to the current body of knowledge, offering specific insights into the effects of exercise on CLL patients. This will not only aid in refining current treatment approaches but also serve as important guidance for future research and clinical practices in this area. Despite the benefits noted, it is also worth mentioning the potential harms induced by the intervention that participants may suffer. Fatigue and exhaustion may be two of the main complications associated with the exercise programme. It will be necessary to individually adjust and optimise the intervention to try to prevent the occurrence of these complications.

### 3.2 Limitations

This study presents three main limitations. First, the progression of the disease itself, as well as tumor-related complications, may result in participant dropout. Second, the presence of comorbidities, whether pre-existing before the cancer diagnosis or arising as a consequence of the disease and its treatment, could affect the standardization of interventions. However, these limitations are inherent to any complex clinical process. Third, the side effects of oncological treatment—such as fatigue, metabolic alterations, and immune function suppression—may impact training capacity and progression. To address this, the study incorporates a flexible approach to workload adjustment based on perceived exertion (RPE ≥7), allowing for load reductions when necessary to optimize safety and adherence (47, 48).

## Data Availability

The original contributions presented in the study are included in the article/Supplementary Material, further inquiries can be directed to the corresponding author.
