# Supplementary material for: Effects of a strength physical exercise program in chronic lymphocytic leukemia patients on quality of life, mental health, and frailty: a randomized controlled trial study protocol
Source: Front Sports Act Living. 2025 Mar 10;7:1534861. doi: 10.3389/fspor.2025.1534861 (PMC11931114; doi:10.3389/fspor.2025.1534861)
Supplement: Supplementary file 1 [file Table1.docx]

The schedule of enrolment, interventions, and assessments of SPIRIT 2013.

|  | **STUDY PERIOD** | | | | | |
| --- | --- | --- | --- | --- | --- | --- |
|  | **Enrolment** | **Allocation** | **Post-allocation** |  | **Close-out** |  |
| **TIMEPOINT** | ***Month 1*** | **Month 2** | ***Month 3*** | ***Month 4*** | ***Month 5-7*** |  |
| **ENROLMENT:** | X |  |  |  |  |  |
| **Eligibility screen** | X |  |  |  |  |  |
| **Informed consent** | X |  |  |  |  |  |
| **Database creation** | X |  |  |  |  |  |
| **Allocation** |  | X |  |  |  |  |
| **INTERVENTIONS:** |  |  |  |  |  |  |
| ***Intervention A: Experimental group (Supervised training sessions)*** |  |  |  |  |  |  |
| ***Intervention B control group***  ***(Physical activity promotion program)*** |  |  |  |  |  |  |
| **ASSESSMENTS:** |  |  |  |  |  |  |
| ***Baseline intervening variables*** | X |  |  |  |  |  |
| ***Fraility, quality of life, anxiety, depression, fatigue, sleep quality, physical activity, body composition, strength and physical performance.*** | X |  |  |  | X |  |
| ***Dissemination of results*** |  |  |  |  | x |  |
