# Supplementary material for: Effects of a strength physical exercise program in chronic lymphocytic leukemia patients on quality of life, mental health, and frailty: a randomized controlled trial study protocol
Source: Front Sports Act Living. 2025 Mar 10;7:1534861. doi: 10.3389/fspor.2025.1534861 (PMC11931114; doi:10.3389/fspor.2025.1534861)
Supplement: Supplementary file 2 [file Table2.docx]

| **Item Category** | **Item No** | **Abbreviated Item Description** |  |
| --- | --- | --- | --- |
| **WHAT:** materials | 1 | Type of exercise equipment | Resistance bands, barbells, and dumbbells. |
| **WHO:** provider | 2 | Qualifications, teaching/supervising expertise, and/or training of the exercise instructor | Physiotherapist with over 7 years of experience in therapeutic exercise prescription and specialized training in oncology. |
| **HOW:** delivery | 3 | Whether exercises are performed individually or in a group | Conducted in group sessions. |
|  | 4 | Whether exercises are supervised or unsupervised | One group supervised in-person, the other monitored via telephone. |
|  | 5 | Measurement and reporting of adherence to exercise | Daily adherence logbook. |
|  | 6 | Details of motivation strategies | Supervised exercise, progressive and individualized training. |
|  | 7 | Decision rules for progressing the exercise program | Increased load if RPE <7. |
|  | 8 | Each exercise is described so that it can be replicated (eg, illustrations, photographs) | No |
|  | 9 | Content of any home program component | Yes |
|  | 10 | Nonexercise components | No |
|  | 11 | How adverse events that occur during exercise are documented and managed | Recorded and reported upon program completion. |
| **WHERE:** | 12 | Setting in which exercises are performed | Therapeutic exercise room and participants' homes. |
| **WHEN, HOW MUCH:** | 13 | Detailed description of the exercises (eg, sets, repetitions, duration, intensity) | 8 weeks, twice a week, initial load 70% 1-RM, three sets of 12 repetitions per exercise, progressive load if RPE <7. |
| **TAILORING:** | 14 | Whether exercises are generic (“one size fits all”) or tailored to the individual | Tailored to the individual |
|  | 15 | Decision rule that determines the starting level for exercise | Exercise ejecution: proper technique maintained, progression if RPE <7. |
| **HOW WELL:** | 16 | Whether the exercise intervention is delivered and performed as planned | Adherence, dropouts, and any deviations from the planned protocol will be recorded. |
